# Pathological complete response in a patient with pleural mesothelioma treated with immunotherapy: a case report

**DOI:** 10.3389/fonc.2024.1378530

**Published:** 2024-04-15

**Authors:** Eleonora Faccioli, Federica Grosso, Andrea Dell’Amore, Sara Delfanti, Giovanni Zambello, Luigi Cerbone, Gianluca Canu, Antonina De Angelis, Viola Sambataro, Federica Pezzuto, Paola Barbieri, Giulia Pasello, Fiorella Calabrese, Federico Rea

**Affiliations:** ^1^ Thoracic Surgery Unit, Department of Cardiac, Thoracic, Vascular Sciences and Public Health, University Hospital of Padova, Padova, Italy; ^2^ Mesothelioma and Rare Cancers Unit, AOU SS Antonio e Biagio e Cesare Arrigo, Alessandria, Italy; ^3^ Pathology Unit, Department of Cardiac, Thoracic, Vascular Sciences and Public Health, University Hospital of Padova, Padova, Italy; ^4^ Pathology, AOU SS Antonio e Biagio e Cesare Arrigo, Alessandria, Italy; ^5^ Department of Surgery, Oncology and Gastroenterology, University of Padova, Padova, Italy; ^6^ Medical Oncology 2, Veneto Institute of Oncology IOV-IRCCS, Padova, Italy

**Keywords:** pleural mesothelioma, surgery, pathological complete response, immunotherapy, multimodal treatment

## Abstract

The role of immunotherapy in the multimodal treatment for pleural mesothelioma (PM) is still under investigation, particularly in the preoperative setting. Pathological complete response (pCR) has been previously described after chemotherapy and immunotherapy; however, there is no prior experience reported with immunotherapy alone before surgery. We report the case of a 58-year-old male with biphasic PM treated with immunotherapy, resulting in a major clinical partial response. Following a multidisciplinary evaluation between thoracic surgeons, medical oncologists, pathologists, radiologists and radiation oncologists, the patient underwent surgery with radical intent through a right extended pleurectomy/decortication (eP/D). Histopathological examination of the specimen confirmed a pathological Complete Response (pCR). This case supports the feasibility and potential efficacy of combining preoperative immunotherapy with surgery in the management of advanced PM.

## Introduction

Pleural mesothelioma is a rare and aggressive disease associated with asbestos exposure, presenting significant challenges in its treatment (1). As of today, when surgical intervention is feasible, the most recommended approach is multimodal, typically involving preoperative platinum-based chemotherapy plus pemetrexed. Subsequently, a surgical procedure (pleurectomy/decortication or extra-pleural pneumonectomy) is performed to achieve a macroscopic complete resection (MCR). This can be followed by adjuvant radiotherapy or immunotherapy as part of clinical trials (1). In recent years, immunotherapy has emerged as a promising treatment for patients affected by unresectable PM, especially for those with non-epithelioid histology (2, 3). Other ongoing trials are evaluating immunotherapy alone or in combination with chemotherapy in the preoperative settings for resectable PM, but this approach has not yet received approval. Here, we present the case of a patient affected by biphasic epithelioid PM with unresectable disease treated with up-front immunotherapy, based on Nivolumab 360 mg + Ipilimumab 80 mg (five cicles). Due to the major radiological response and patient’s good performance status, after multidisciplinary discussion between thoracic surgeons, medical oncologists, pathologists, radiologists and radiation oncologists and adequate patient information, we decided to proceed with radical surgery.

The patient has expressed a written informed consent to be involved in clinical studies.

## Case description

**Figure 1:**
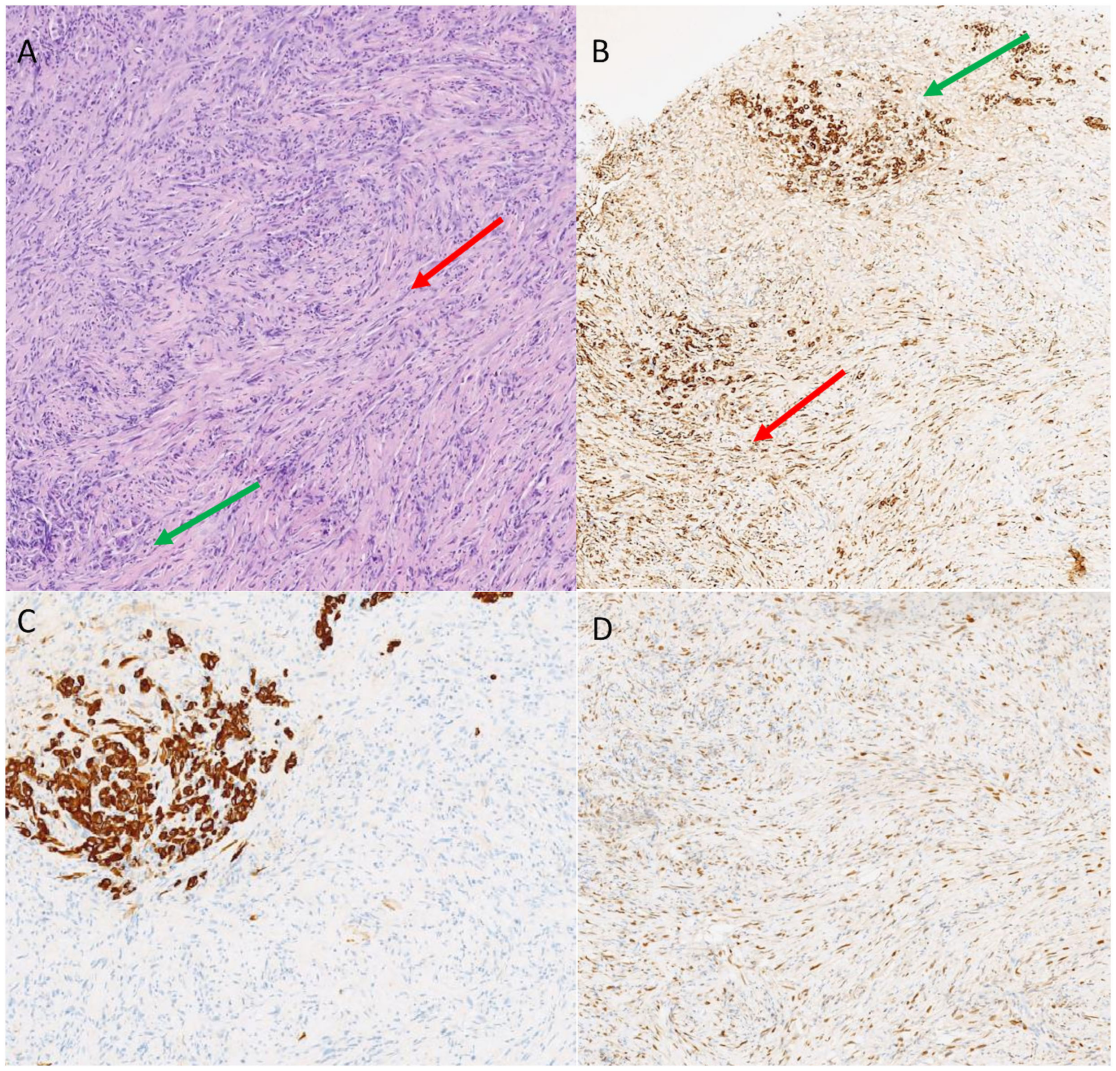
Hematoxylin and eosin staining identified both the epitelioid (green arrow) and sarcomatoid (red arrow) components **(A)**. Immunostaining for pancytokeratin was positive for both the components (green arrow, epithelioid; red arrow, sarcomatoid, **B**). CK5 identified the epithelioid component **(C)** while GATA 3 the sarcomatoid **(D)**.

**Figure 2:**
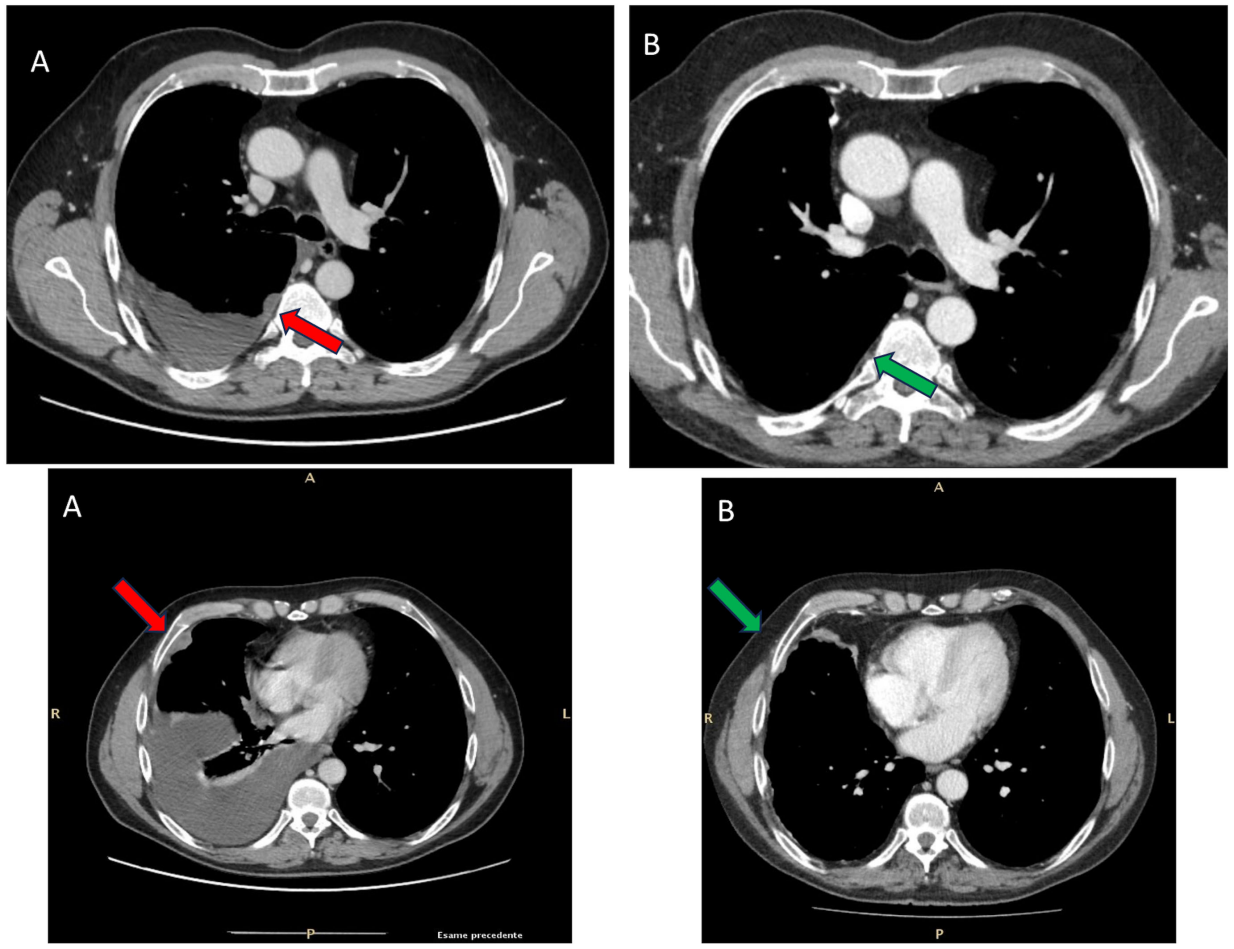
Chest computed tomography scan of the patient before **(A)** and after four cycles of immunotherapy (nivolumab and ipilimumab) **(B)**. In image A, the red arrow indicates the significant pleural thickening, while in B, the green arrow highlights the substantial reduction in the disease burden.

**Figure 3:**
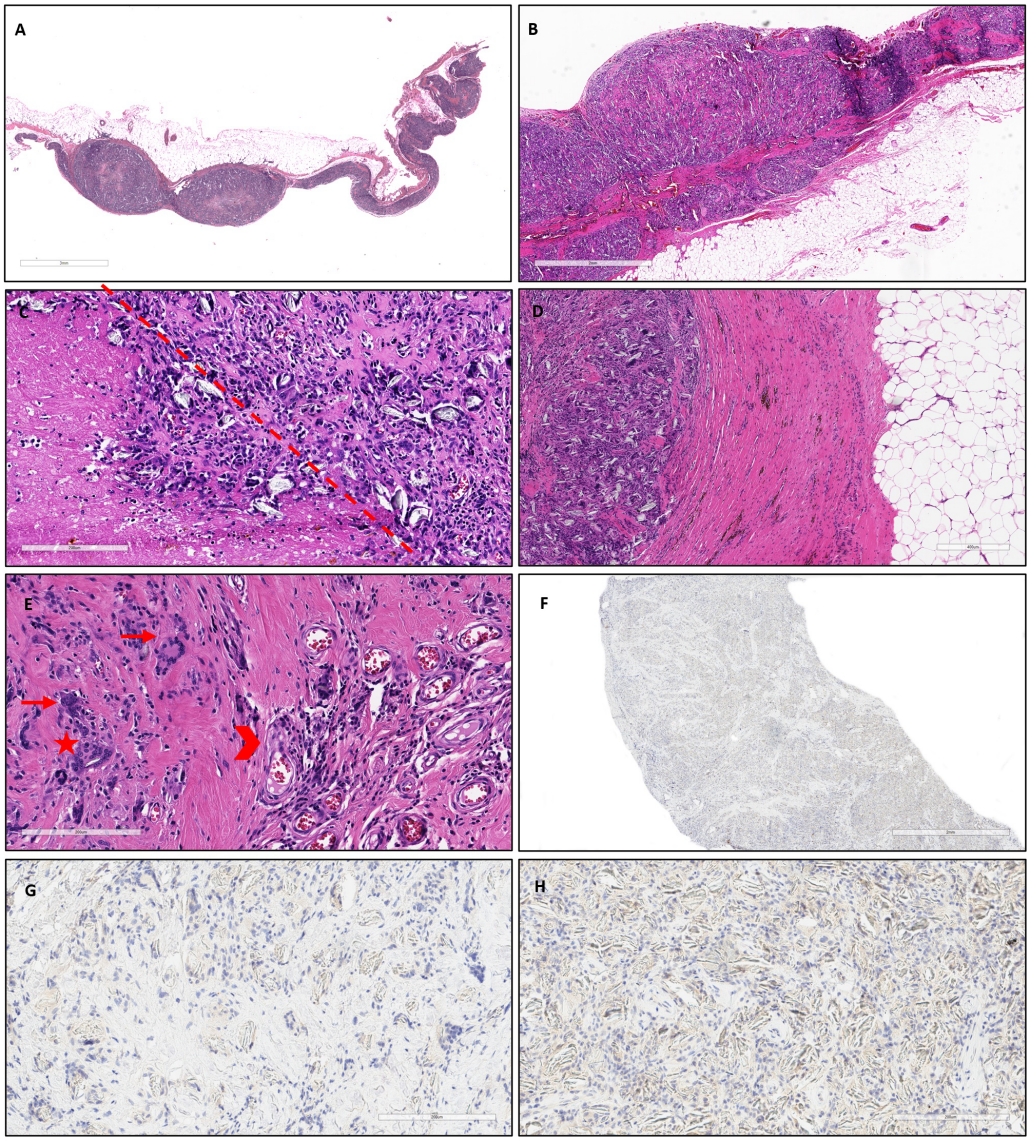
At low magnification, the pleura was diffusely thickened with some nodular structures (**A**, hematoxylin and eosin, scale bar: 3 mm) and well demarcated with respect to the subpleural fat tissue (**B**, hematoxylin and eosin, scale bar: 2 mm). At higher magnification, several foci of necrosis were detected within the nodules (left side of the dot-line), alongside granulomatous foreign body inflammation (**C**, hematoxylin and eosin, scale bar: 200 µm). The granulomatous component was separated from the underlying adipose tissue by intense fibrosis, with lymphomonocytic inflammation and focal hemosiderin deposition (**D**, hematoxylin and eosin, scale bar: 400 µm). Additionally, fibrosis, neoangiogenesis (arrowhead), and a predominant infiltration of multinucleated cells (arrows) with some cholesterol clefts (star) were observed (**E**, hematoxylin and eosin, scale bar: 200 µm). Immunostaining for cytokeratin, for detecting scattered tumor cells (MNF116) was negative (**F**, scale bar: 2 mm; **G**, **H**: scale bar: 200 µm).

A 58-year-old male patient, former smoker (30 pack/year) with an history of environmental asbestos exposure, presented with pain in the right hypochondrium along with cough and exertional dyspnea. A chest X-ray revealed right-pleural effusion. A subsequent computed tomography (CT)-scan showed diffuse thickening of the right pleural with nodular components, accompanied by pleural effusion. PET-CT scan revealed diffuse pathological uptake in the right pleura but showed no evidence of metastatic disease in other sites. The patient underwent a CT-guided pleural biopsy with the diagnosis of epithelioid PM but since the histotype was not consistent with the radiological aspect of the disease, the patient underwent pleural biopsies and chemical pleurodesis through video-assisted thoracic surgery (VATS). According to current guidelines (1), based on morphology and immunohistochemistry, the final diagnosis confirmed a biphasic mesothelioma, with a minor (less than 5%) component of spindle cells (**Figure 1**). Due to the histology and the high disease burden (mRECIST at diagnosis 139 mm), the patient was deemed unresectable. Following guidelines, immunotherapy with Nivolumab 360 mg and Ipilimumab 80 mg was prescribed and administered for five courses. Mild diarrhea, related to the treatment, and episodes of atrial fibrillation, likely related to the proximity of the disease to the pericardium, were the only reported adverse events during treatment. The CT scan performed after the second course showed consistent tumor shrinkage (mRECIST 15 mm), a finding confirmed at the subsequent tumor reassessment after the fourth course (mRECIST 14 mm) (**Figure 2**). Considering the significant radiological partial response, the patient’s age, and good performance status, a multidisciplinary discussion of the case and in-depth discussion with the patient, deemed him eligible for surgery. A right extended pleurectomy/decortication with mediastinal lymph node dissection was performed. After sampling the entire specimen and conducting serial sections for each block, histological examination revealed diffusely thickened pleura with nodular structures, well-demarcated from subpleural fat tissue. Foci of necrosis within the nodules, alongside granulomatous inflammation, were observed at higher magnification. Intense fibrosis, lymphomonocytic inflammation, and focal hemosiderin deposition were evident, along with fibrosis, neoangiogenesis, and infiltration of multinucleated cells with cholesterol clefts. Immunostaining for cytokeratin (MNF116) was negative. Based on these findings, a pathological complete response (pCR) was reported (**Figure 3**). The postoperative course was uneventful, and the patient was discharged in good clinical conditions after eleven days. At six months after surgery, the patient is alive with no signs of recurrence and metastasis.

## Discussion

We present a case of pCR in a patient with a biphasic PM who underwent eP/D following immunotherapy. Optimal treatment for PM requires thorough discussion within a multidisciplinary expert team and should only be undertaken in highly specialized centers. The precise histology on well representative samples is crucial to establish the optimal treatment approach: according to clinical practice guidelines (1) thoracoscopy is recommended to obtain adequate histology with deep biopsies from ideally three pleural sites and CT scan or ultrasound-guided biopsies should be considered good alternatives when thoracoscopy is not feasible or contraindicated. Patients with good performance status, non-sarcomatoid histology, and early-stage disease may be suitable for surgery as part of a multimodal treatment which invariably always includes chemotherapy and may also involve radiotherapy in selected cases (1, 4). In PM, clinical studies investigating the use of immune checkpoint inhibitors (ICIs) or chemotherapy (CT) combined with ICIs are currently ongoing. The only evidence regarding the use of PD-L1 CTLA4 blockade comes from a phase II window of opportunity trial primarily focused on evaluating intra-tumoral T cell infiltrates and tumor microenvironment (5). Ongoing trials, such as one associating pembrolizumab with defactinib (NCT04201145) and nivolumab with pemetrexed plus platin, (NCT04162015), are exploring these avenues.

In our case, the decision to start immunotherapy as first-line treatment was based on several aspects: first of all, as reported in the Checkmate 743, the response rate and the overall survival are significantly higher with immunotherapy rather than chemotherapy in non-epithelioid subtypes (3). Furthermore, due to regulatory rules, in case of no response to immunotherapy, we would still have the possibility to administer a second-line chemotherapy but in case of starting chemotherapy, the patient would have never had the possibility to receive immunotherapy. Giving the major partial radiological response, the young age and the good performance status, we decided to submit the patient to surgery with radical intent, through an extended pleurectomy/decortication, to achieve macroscopic complete resection of the disease.

Limited experiences with these agents before surgery are currently documented in short reports. Tostes et al. (6) reported a pCR in a patient with epithelioid PM who underwent surgery after neoadjuvant therapy with cisplatin, pemetrexed, and off-label pembrolizumab. While the concept of resectable PM is currently under debate (7), the utilization of ICIs or ICIs combined CT in the pre-operative setting holds promise for new therapeutic opportunities in PM. It is essential to note that, due to the rarity of this disease, the expertise of the center and diagnostic accuracy are crucial factors for achieving the best response.

In conclusion, we present the first case of a pCR in a patient with PM previously treated with nivolumab and ipilimumab. This case highlights the potential benefits of immunotherapy in a neoadjuvant setting for PM. Further studies are mandatory to enhance the pre-operative experience with ICIs, either alone or in combination with CT, and to better understand the optimal settings for utilizing these agents.

## Data availability statement

The original contributions presented in the study are included in the article/supplementary material. Further inquiries can be directed to the corresponding author.

## Ethics statement

Due to the nature of the study (case report) ethical approval was waived. The studies were conducted in accordance with the local legislation and institutional requirements. The participants provided their written informed consent to participate in this study. Written informed consent was obtained from the individual(s) for the publication of any potentially identifiable images or data included in this article. Written informed consent was obtained from the participant/patient(s) for the publication of this case report.

## Author contributions

EF: Writing – original draft, Writing – review & editing. FG: Writing – original draft, Writing – review & editing. AD: Conceptualization, Validation, Writing – review & editing. SD: Investigation, Supervision, Writing – review & editing. GZ: Data curation, Investigation, Writing – review & editing. LC: Supervision, Validation, Writing – review & editing. GC: Data curation, Investigation, Writing – review & editing. ADA: Supervision, Validation, Writing – review & editing. VS: Data curation, Investigation, Writing – review & editing. FP: Data curation, Investigation, Validation, Writing – review & editing. PB: Conceptualization, Supervision, Validation, Writing – review & editing. GP: Conceptualization, Supervision, Validation, Writing – review & editing. FC: Conceptualization, Supervision, Validation, Writing – review & editing. FR: Conceptualization, Supervision, Validation, Writing – review & editing.

## References

[1] Popat S Baas P Faivre-Finn C Girard N Nicholson AG Nowak AK. Malignant pleural mesothelioma: ESMO Clinical Practice Guidelines for diagnosis, treatment and follow-up. Ann Oncol. (2022) 33:129–42. doi: 10.1016/j.annonc.2021.11.005 34861373

[2] Nowak AK Lesterhuis WJ Kok PS Brown C Hughes BG Karikios DJ. Durvalumab with first-line chemotherapy in previously untreated Malignant pleurale mesothelioma (DREAM). A multicentre, single-arm, phase 2 trial with safety run-in. Lancet Oncol. (2020) 21:1213–23. doi: 10.1016/S1470-2045(20)30462-9 32888453

[3] Baas P Scherpereel A Nowak A Fujimoto N Peters S Tsao AS. First-line nivolumab plus ipilimumab in unresectable Malignant pleural mesothelioma (CheckMate 743):a multicentre, randomized, open-label, phase 3 trial. Lancet. (2021) 397:385–6. doi: 10.1016/S0140-6736(20)32714-8 33485464

[4] Pasello G Ceresoli GL Favaretto A. An overview of neoadjuvant chemotherapy in the multimodality treatment of Malignant pleural mesothelioma. Cancer Treat Rev. (2013) 39:10–7. doi: 10.1016/j.ctrv.2012.03.001 22459200

[5] Lee HS Jang HJ Ramineni M Wang DY Ramos D Choi JM. A Phase II Window of Opportunity Study of Neoadjuvant PD-L1 versus PD-L1 plus CTLA-4 Blockade for Patients with Malignant Pleural Mesothelioma. Clin Cancer Res. (2023) 29:548–59. doi: 10.1158/1078-0432.CCR-22-2566 PMC9898180 36469573

[6] Tostes FT Zugman M Paes VR Schvartsman G. Complete pathological response after neoadjuvant chemo-immunotherapy in Malignant pleural mesothelioma. Front Oncol. (2022) 12:836751. doi: 10.3389/fonc.2022.836751 35574305 PMC9096134

[7] Lim E Waller D Lau K Steele J Pope A Ali C. PL03.10 MARS 2: A multicentre randomised trial comparing (Extended) pleurectomy decortication versus no radical surgery for mesothelioma. J Thorac Oncol. (2023) 18:S36. doi: 10.1016/j.jtho.2023.09.008

